# Ocular Dominance Plasticity after Stroke Was Preserved in PSD-95 Knockout Mice

**DOI:** 10.1371/journal.pone.0149771

**Published:** 2016-03-01

**Authors:** Franziska Greifzu, Daniel Parthier, Bianka Goetze, Oliver M. Schlüter, Siegrid Löwel

**Affiliations:** 1 Systems Neuroscience, Bernstein Fokus Neurotechnologie, Johann-Friedrich-Blumenbach-Institut für Zoologie und Anthropologie, Georg-August-Universität, Göttingen, Germany; 2 European Neuroscience Institute Göttingen, Göttingen, Germany; University of Nebraska Medical Center, UNITED STATES

## Abstract

Neuronal plasticity is essential to enable rehabilitation when the brain suffers from injury, such as following a stroke. One of the most established models to study cortical plasticity is ocular dominance (OD) plasticity in the primary visual cortex (V1) of the mammalian brain induced by monocular deprivation (MD). We have previously shown that OD-plasticity in adult mouse V1 is absent after a photothrombotic (PT) stroke lesion in the adjacent primary somatosensory cortex (S1). Exposing lesioned mice to conditions which reduce the inhibitory tone in V1, such as raising animals in an enriched environment or short-term dark exposure, preserved OD-plasticity after an S1-lesion. Here we tested whether modification of excitatory circuits can also be beneficial for preserving V1-plasticity after stroke. Mice lacking postsynaptic density protein-95 (PSD-95), a signaling scaffold present at mature excitatory synapses, have lifelong juvenile-like OD-plasticity caused by an increased number of AMPA (α-amino-3-hydroxy-5-methyl-4-isoxazolepropionic acid) -silent synapses in V1 but unaltered inhibitory tone. In fact, using intrinsic signal optical imaging, we show here that OD-plasticity was preserved in V1 of adult PSD-95 KO mice after an S1-lesion but not in PSD-95 wildtype (WT)-mice. In addition, experience-enabled enhancement of the optomotor reflex of the open eye after MD was compromised in both lesioned PSD-95 KO and PSD-95 WT mice. Basic V1-activation and retinotopic map quality were, however, not different between lesioned PSD-95 KO mice and their WT littermates. The preserved OD-plasticity in the PSD-95 KO mice indicates that V1-plasticity after a distant stroke can be promoted by either changes in excitatory circuitry *or* by lowering the inhibitory tone in V1 as previously shown. Furthermore, the present data indicate that an increased number of AMPA-silent synapses preserves OD-plasticity not only in the healthy brain, but also in another experimental paradigm of cortical plasticity, namely the long-range influence on V1-plasticity after an S1-lesion.

## Introduction

If the brain is injured, for example due to a stroke lesion, neuronal plasticity is crucial for recovery and to promote rehabilitation. The primary visual cortex (V1) is a widely used model region for studying experience-dependent plasticity in the mammalian brain [1, 2]. Neurons in the binocular zone of rodent V1 respond to stimulation of both eyes but are dominated by the contralateral eye [3, 4]. This ocular dominance (OD) can be modified in an experience-dependent manner by depriving one eye of pattern vision for several days (known as monocular deprivation or MD), as originally observed by Wiesel and Hubel more than 50 years ago in cat V1 [5]. In a previous study, we observed that a small photothrombotic (PT) stroke lesion in S1, directly adjacent to V1, or even in M2, nearly 4 mm anterior to the anterior border of V1, abolished OD-plasticity in V1 and impaired experience-enabled improvements of the optomotor reflex of the open eye after MD in adult mice [6, 7].

While OD-plasticity in standard cage raised rodents is limited by age [1, 2], several interventions have been recently published to promote plasticity in V1 of adult rodents [8]. These plasticity promoting interventions include housing animals in an enriched environment [9–11], short-term dark exposure [12–14], running [15], running and visual stimulation [16], strong visual stimulation [17], and previous MD [18]. Most of these interventions likely function via reducing intracortical inhibition. Correspondingly, reducing cortical inhibition pharmacologically in adult rats also promoted OD-plasticity [19].

Notably, enriched environment or short-term dark exposure have previously been shown to also preserve OD-plasticity in V1 after an S1-lesion [9, 12]. Whether changes in excitatory circuitry may also be beneficial for preserving V1-plasticity after a stroke in S1 was—however—not yet tested.

Mice lacking the postsynaptic signaling scaffold PSD-95 have recently been shown to display a lifelong and juvenile-like OD-plasticity, caused by an increased number of AMPA (α-amino-3-hydroxy-5-methyl-4-isoxazolepropionic acid) -silent synapses, but unaltered inhibitory tone in V1 [20]. These new data clearly indicated that inhibitory circuits may play a permissive role for cortical plasticity while silent synapse maturation is critical and rather instructive: the sensitive phase for OD-plasticity did not close in the absence of PSD-95, even if the inhibitory tone matured normally, and OD-plasticity was also restored if PSD-95 was knocked-down in the adult visual cortex [20]. The essential role of silent synapses for OD-plasticity caused a paradigm shift in the way we think about critical period closure in the adult brain. It is therefore important to test whether other experimental paradigms of cortical plasticity such as the long-range influence on OD-plasticity in V1 by a distant lesion share similar mechanisms or are distinct.

We therefore tested whether knockout (KO) mice for PSD-95 display a preserved OD-plasticity in V1 after a small lesion in S1, adjacent to V1. To this end, we induced a photothrombotic stroke lesion in S1, about 1 mm anterior to the anterior border of V1 and visualized OD-plasticity using *in vivo* optical imaging of intrinsic signals. Additionally, we measured both the spatial frequency and contrast thresholds of the optomotor reflex of the open eye after MD by optomotry. In fact, OD-plasticity after an S1-lesion was preserved in adult PSD-95 KO mice but not in PSD-95 WT mice. In addition, experience-enabled changes of the optomotor reflex were compromised in both S1-lesioned PSD-95 KO and PSD-95 WT mice after MD.

## Material and Methods

### Animal treatment

Adult female and male PSD-95 KO and WT mice were used [20, 21]. 2–5 mice were housed together in standard cages (32 × 16 × 14 cm) with a 12-h light/dark cycle, food and water available *ad libitum*. The indicated age is the age at the optical imaging experiment.

**Ethics statement:** All experimental procedures were performed according to the German Law on the Protection of Animals and permitted by the local government: Niedersächsisches Landesamt für Verbraucherschutz und Lebensmittelsicherheit and approved by the Tierschutz Kommission des Landes Niedersachsen § 15 TSG (Permission no 33.9-42502-04-10/0326 and 33.9-42502-04-14/1516).

### Monocular deprivation (MD)

The right eye was deprived for 7 days according to published protocols [6, 22, 23]. Briefly, mice were anesthetized with 2% isoflurane in 1:1 O_2_:N_2_O. Lid margins were trimmed and an antibiotic gel (gentamicin gel) was applied. The eye was closed with two mattress sutures. Animals were checked daily to make sure that the eyes remained closed.

### Spatial frequency and contrast sensitivity threshold of the optomotor reflex

Both the spatial frequency and the contrast threshold of the optomotor reflex of all mice was measured daily using the virtual-reality optomotor system developed by Prusky et al. [24]. Briefly, freely moving animals were exposed to moving sine wave gratings of various spatial frequencies and contrasts and reflexively track the gratings by head movements as long as they can see the gratings. Spatial frequency at full contrast and contrast at six different spatial frequencies [0.031, 0.064, 0.092, 0.103, 0.192, 0.272cycles/degree (c/d)] were varied by the experimenter until the threshold of tracking was determined. Contrast sensitivity thresholds of the optomotor reflex measured in percent were converted into Michelson contrasts. All animals were first tested in the optomotor setup and then their visual cortical activity was analyzed by optical imaging of intrinsic signals.

### Optical imaging of intrinsic signals and visual stimuli

Following the behavioral tests, mouse visual cortical responses were recorded and analyzed as described previously [6].

#### Surgery

Briefly, mice were box-anesthetized with 2% halothane in a mixture of O_2_:N_2_O (1:1) and received an injection of atropine (Franz Köhler, 0.3 mg/mouse, subcutaneously), dexamethasone (Ratiopharm, 0.2 mg/mouse, subcutaneously), and chlorprothixene (Sigma, 0.2 mg/mouse, intramuscularly). After placing the animals in a stereotaxic frame, inhalation anesthesia was maintained with 0.6% to 0.8% halothane in a mixture of O_2_:N_2_O (1:1). The animals’ body temperature was maintained at 37°C and heart rate was monitored throughout the experiment.

#### Data acquisition and visual stimulation

Mouse visual cortical responses were recorded through the skull using the imaging method developed by Kalatsky and Stryker [25] and optimized for the assessment of OD-plasticity by Cang et al. [23]. In this method, a temporally periodic stimulus is continuously presented to the animal and the cortical response at the stimulus frequency is extracted by Fourier analysis. Optical images of intrinsic cortical signals were obtained using a CCD camera (Dalsa 1M30) controlled by custom software. The surface vascular pattern and intrinsic signal images were visualized with illumination wavelengths set by a green (550±10 nm) or red (610±10 nm) interference filter, respectively. After acquisition of a surface image, the camera was focused 600 μm below the cortical surface. An additional red filter was interposed between the brain and the CCD camera. Frames were acquired at a rate of 30 Hz temporally binned to 7.5 Hz and stored as 512 x 512 pixel images after spatial binning of the camera image. Drifting horizontal bars (2° wide) were presented to the animal at a distance of 25 cm on a high refresh-rate monitor (Hitachi ACCUVUE, HM-4921-D, 21”), positioned 25 cm from the eyes. The distance between two bars was 80° and they were presented at a temporal frequency of 0.125 Hz. The visual stimulus was restricted to the binocular visual field of the left V1 (−5° to +15° azimuth) and animals were stimulated through either the left or the right eye in alternation to assess the OD of the left hemisphere. For visualizing elevation and azimuth maps for map quality calculation, we used full-field stimuli extending 78° horizontally and 59° vertically and contralateral eye stimulation.

#### Data analysis

Visual cortical maps were calculated from the acquired frames by Fourier analysis to extract the signal at the stimulation frequency using custom software [25]. The amplitude component represents the intensity of neuronal activation (expressed as fractional change in reflectance ×10^−4^) and was used to calculate OD. At least three maps per animal were averaged to compute the OD-index (ODI) as (C—I)/(C + I), with C and I representing the response magnitudes of each pixel to visual stimulation of the contralateral (C) and ipsilateral (I) eye. The ODI ranged from −1 to +1, with negative values representing ipsilateral and positive values representing contralateral dominance. ODI-data of sham-treated and PT-lesioned (S1) C57Bl6/J (WT) mice are from Greifzu et al. [6], but newly analyzed as described above (“average map analysis”).

The quality of the retinotopic maps was assessed by the calculation described by Cang et al. [26] on contralateral eye full field maps. For each of the pixels in the retinotopic map, the difference between its visual field position and the mean position of its surrounding 24 pixels was calculated. For maps of high quality, the position differences are quite small because of smooth progression. The standard deviation of the position difference was then used as an index of the quality of retinotopic maps with a small standard deviation, which corresponds to low map scatter, indicating high map quality and high values, corresponding to high map scatter, indicating low map quality.

### Induction of photothrombosis

A photothrombotic lesion was induced in the left S1 adjacent to V1 by using the Rose Bengal technique introduced by Watson et al. [27], as described previously [6]. Mice were initially anesthetized with 2% isoflurane, and anesthesia was maintained with 1% isoflurane in 1:1 O_2_:N_2_O. The animals were placed in a stereotaxic frame and body temperature was maintained at 37°C. The skin above the skull was incised and an optic fiber bundle (aperture: 1.0 mm) mounted on a cold light source (Schott KL 1500) was positioned 2 mm lateral to the midline and 1 mm posterior to the bregma. Next, 100 μl Rose Bengal (Aldrich; 10 mg/ml in 0.9% NaCl) was injected intravenously. After 5 minutes, the illumination period of 15 minutes was started. The skin was sutured and the animals recovered in their cage.

### Perfusion and tissue processing

After optical imaging, all mice were perfused transcardially with 1% heparin in 0.9% NaCl for 2 minutes followed by 4% paraformaldehyde (PFA, pH 7.4) for 3 minutes. The brain was removed and postfixed in 4% PFA (pH 7.4) for one day and then transferred to cryoprotectant solution (10% sucrose, 20% glycerol). The brains were frozen in methylbutane and stored at -80°C. Coronal brain sections were cut on a sledge microtome at 40 μm.

### Lesion analysis

To determine size and location of the cortical PT-lesions, coronal brain sections were Nissl-stained and every third section was analyzed under the microscope (Axioskop, Carl Zeiss). Quantitative parameters were measured using AxioVision (40 4.8.2.0.).

### Statistical analyses

All intra- and intergroup comparisons were analyzed by a two-tailed Student's t-test (with Bonferroni correction). The levels of significance were set as *P<0.05; **P<0.01; ***P<0.001. Data are represented as means±SEM.

## Results

### Location and size of the cortical lesion in the primary somatosensory cortex (S1)

**Fig 1:**
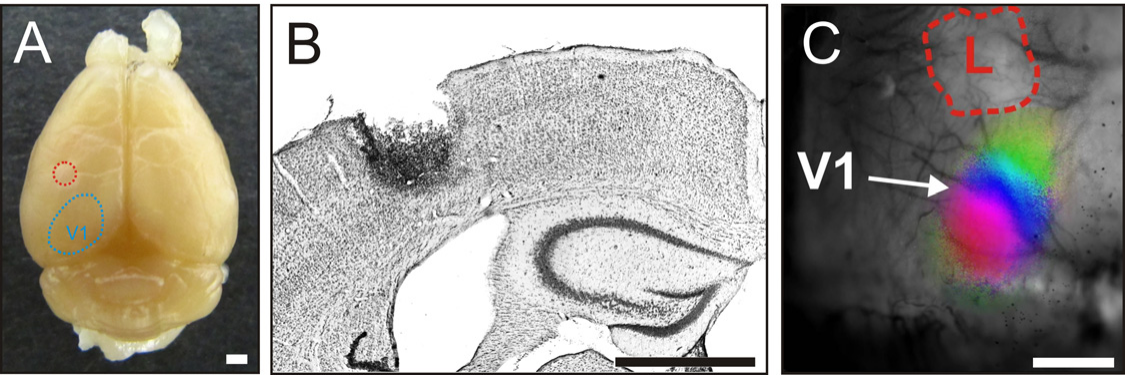
Location of the photothrombotically induced cortical stroke lesion in a PSD-95 KO mouse in S1 (PT, red dashed line). (A) Top view of a representative mouse brain illustrating the lesion location in S1, on average 1 mm anterior to the anterior border of the primary visual cortex (V1, blue dashed line). (B) Nissl-stained frontal section through the lesion (same animal as in A). (C) Higher magnification composite image of the superficial vascular pattern of the brain and the superimposed optically recorded retinotopic map of the binocular part of V1 of a PSD-95 KO mouse in which the PT-lesion (L) was very close to V1; nevertheless, OD-plasticity was present in this animal (average ODI = 0.00). Scale bar all, 1 mm.

The PT-lesions were always located in the left S1 (Fig 1). The lesion in PSD-95 WT/KO mice was on average 0.8±0.1/1.0±0.2 mm in medio-lateral and 0.7±0.1/1.0±0.2 mm in anterior-posterior direction, and extended vertically until layers 5/6. The lesion center was situated 0.8±0.1/1.1±0.1 mm anterior to the anterior border of V1, 2.2±0.2/2.3±0.1 mm lateral to the midline, and 1.4±0.2/1.1±0.2 mm posterior to the bregma. The lesion extent and location were not different between PSD-95 WT and PSD-95 KO mice (P>0.05). Moreover, the lesion values of the KO mice were not different from the previously published values in WT mice [6] which showed diminished plasticity (P>0.05 for all measured parameters).

### In adult PSD-95 KO mice, ocular dominance plasticity in V1 was preserved after a stroke lesion in S1

**Fig 2:**
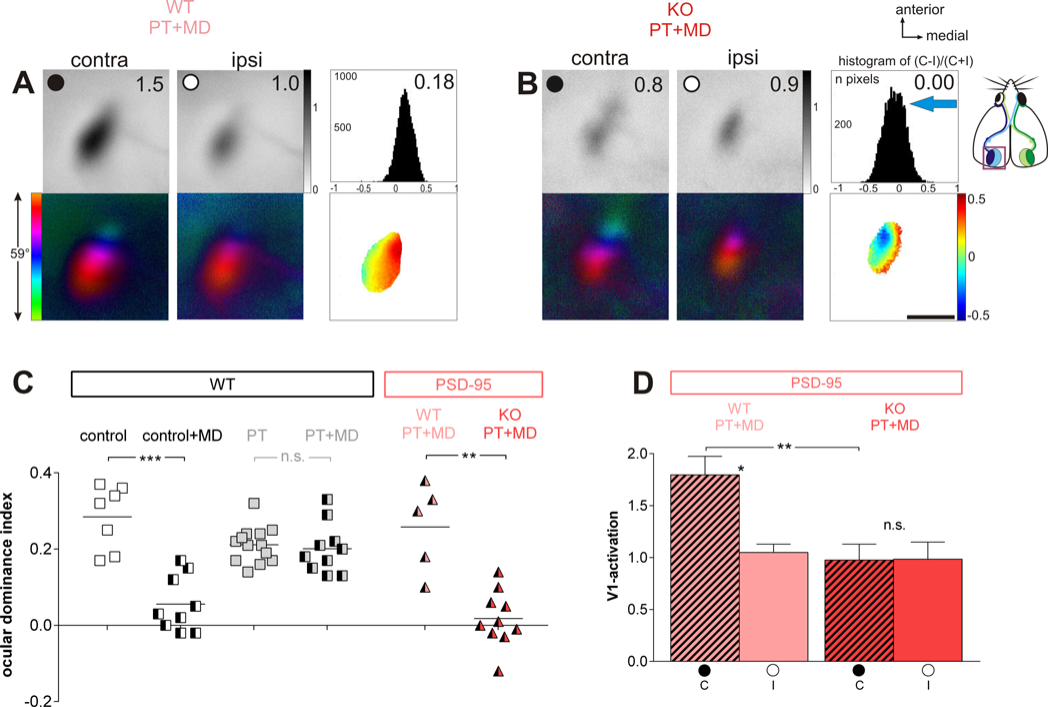
In adult PSD-95 KO mice, ocular dominance plasticity was preserved after a PT-stroke in S1. Optically recorded activity maps of the contralateral (contra) and ipsilateral (ipsi) eye in the binocular region of mouse primary visual cortex (V1) in PT-lesioned PSD-95 WT (A) and PSD-95 KO mice (B) after monocular deprivation (MD), and their quantification (C, D). Open and closed eyes indicated by a white or black circle. (A, B) Grayscale coded response magnitude maps of the contra- and ipsilateral eye (including average V1-activation of the illustrated example) and the histogram of OD-scores including the average OD-index (top row), color-coded polar maps of retinotopy and 2-dimensional OD-maps after MD (lower row) are illustrated. (A) In PSD-95 WT littermates, activity patches evoked by stimulation of the contralateral eye were darker than those of the ipsilateral eye, the average ODI was positive, and warm colors prevailed in the OD-maps, indicating contralateral dominance. In contrast, in PSD-95 KO mice, the contra- and ipsilateral eye activated V1 about equally strong, colder colors appeared in the OD-map, and the histogram of OD-scores shifted to the left (B). Scale bar: 1 mm. (C) Optically imaged OD-indices in PT-lesioned PSD-95 WT and PSD-95 KO mice (light red and red triangles). For comparison, ODIs of control (sham-treated) and PT-lesioned Bl6/J mice are illustrated (white and grey squares). Symbols represent ODI values of individuals, means are marked by horizontal lines. (D) V1-activation elicited by stimulation of the contralateral (C) or ipsilateral (I) eye in PSD-95 WT and PSD-95 KO mice after PT and MD. Note that V1-activtation after stimulation of the contra- and ipsilateral eye was not different in PSD-95 KO mice, indicating preserved juvenile-like OD-plasticity in V1 despite the S1-lesion.

In S1-lesioned PSD-95 WT mice, OD-plasticity in V1 was absent, as previously described for WT mice [6]. After 7 days of MD, activity maps in the binocular zone of V1 were still dominated by the previously deprived, contralateral eye (Fig 2A): activity patches induced by visual stimulation of the contralateral eye were always darker than those after ipsilateral eye stimulation, the average ODI was positive, and warm colors prevailed in the 2‐dimensional OD-maps (Fig 2A). In contrast, PSD-95 KO mice with a PT-lesion in S1 showed an almost equal V1-activation after right and left eye stimulation in the binocular zone after MD, indicating preserved OD-plasticity: the activity spot induced by stimulation of the open (ipsilateral) eye was as dark as the spot induced by stimulation of the deprived (contralateral) eye, colder colors appeared in the OD‐map and the ODI‐histogram was shifted to the left (Fig 2B).

Quantitative analyses of V1-activation revealed that the average ODI of the PSD-95 KO mice was 0.02±0.02 (n = 10, PD93-149), and thus significantly lower than the 0.26±0.05 of the PSD-95 WT mice (n = 5, p = 0.006, t-test; PD78-108; Fig 2C). Although the PT-lesion was very close to V1 in one of the PSD-95 KO mice (Fig 1C), this animal still showed OD-plasticity (ODI = 0.00). In fact, ODI values of PSD-95 KO mice after stroke and MD were in the range of nonlesioned sham-treated (control) WT mice (ODI = 0.06±0.02, n = 9, p = 0.279); compare control+MD with KO PT+MD in Fig 2C) and significantly lower than in nonlesioned sham-treated WT mice without MD (control, ODI = 0.28±0.03, n = 7; p = 0.00002). ODI values of PSD-95 WT mice after stroke and MD stayed as high as in C57Bl6/J WT mice with stroke (C57Bl6/J WT with PT: 0.21±0.01, n = 13; with PT+MD: 0.20±0.02, n = 10; p = 0.425; 0.348).

Analyzing V1-activation induced by left and right eye stimulation separately (Fig 2D) after PT and MD showed that in PSD-95 WT mice, V1-activation after stimulation of the contralateral (right) eye (1.80±0.18) was significantly higher than after ipsilateral (left) eye stimulation (1.05±0.08, n = 5; p = 0.013, t-test). In contrast, in PSD-95 KO mice, V1-activation was the same after contralateral (0.98±0.15) and ipsilateral eye stimulation (0.98±0.16, n = 10; p = 0.906, t-test). Furthermore, the lower V1-activation after deprived eye stimulation indicate that the OD-shift in the PSD-95 KO mice was mediated by a reduction in deprived eye responses in V1 (p = 0.026, t-test), a hallmark of juvenile OD-plasticity.

Note that some of the KO mice with persistent OD-plasticity after stroke were already over 110 days old (n = 8) and therefore already beyond the sensitive period for OD-plasticity in standard cage raised WT mice [28]. Furthermore, there was no correlation of the ODI and the age of the animals (Pearson-correlation -0.080, p = 0.826).

In summary, unlike PSD-95 WT mice, PSD-95 KO mice maintained a juvenile-like OD-plasticity in V1 despite a photothrombotic stroke lesion in S1 and an already advanced age.

### Retinotopic maps and V1-activation were not different in WT and PSD-95 KO mice after stroke

To analyze baseline V1-activation in the PSD-95 WT (n = 5) and KO mice (n = 6) after the S1-lesion, we used full-field horizontal and vertical moving stimuli and recorded retinotopic and activity maps in V1 after contralateral eye stimulation.

For both genotypes, elevation (stimulation with horizontal bars) and azimuth (vertical bars) maps were neither significantly different in signal strength nor in retinotopic map quality. V1-activation of the elevation maps was 2.8±0.3 for WT mice and 1.9±0.4 for KO mice (p = 0.099, t-test). Likewise, V1-responses after azimuth stimulation were also similar (WT/KO: 1.9±0.2/1.9±0.5; p = 0.949, t-test). The quality of the retinotopic maps was also similar: mean map scatter of the elevation maps was 4.9±2.0 for WT mice and 10.9±4.1 for KO mice (p = 0.229, t-test); map scatter for azimuth maps was 16.3±3.1/15.9±3.6 for WT/KO (p = 0.934, t-test).

### The S1-lesion compromised the experience-enabled enhancement of the optomotor reflex of the open eye in both WT and PSD-95 KO mice

**Fig 3:**
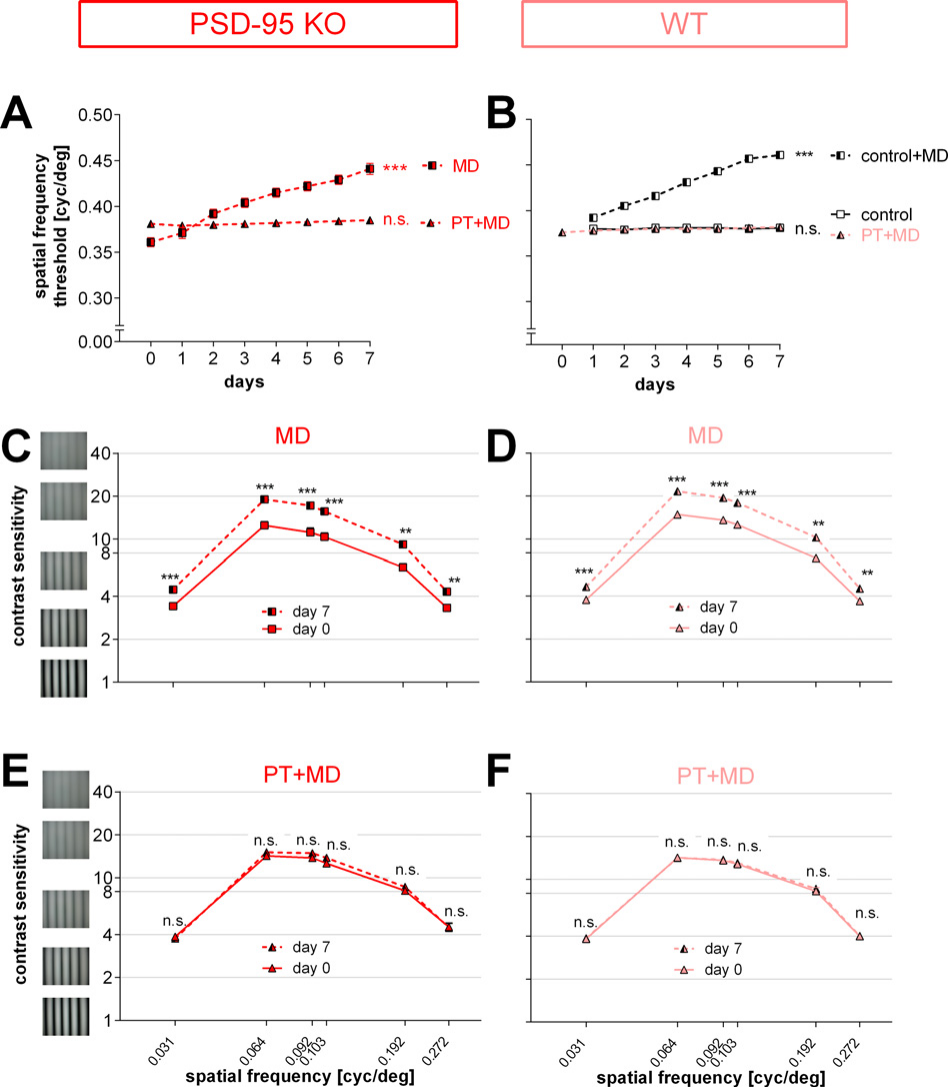
The experience-enabled enhancement of the optomotor reflex of the open eye after monocular deprivation (MD) was compromised in both PT-lesioned PSD-95 WT and PSD-95 KO mice. In contrast, enhancements of spatial vision were present in nonlesioned PSD-95 WT and PSD-95 KO mice. (A, B) Spatial frequency threshold of the optomotor response of the open eye in cycles per degree (cyc/deg) plotted against days after MD. After 7 days of MD, nonlesioned PSD-95 KO mice (A) as well as sham-treated control mice (B; data from Greifzu et al., 2011) showed a significant increase in the spatial frequency threshold of the optomotor reflex of the open eye. This experience-enabled increase was abolished by a PT in S1 (A, B). (C-F) Contrast sensitivity thresholds of the optomotor reflex of the open eye at 6 different spatial frequencies before (day 0) and 7 days after MD. For both nonlesioned PSD-95 KO (C) and PSD-95 WT mice (D), there was an increase in contrast sensitivity after 7 days of MD. After PT, this experience-enabled increase was absent in both groups (E, F).

Spatial vision of all mice was measured during the 7 days of MD (before the optical imaging experiment) using the virtual‐reality optomotor setup [24]. The highest spatial frequency that elicited an optomotor reflex in the animals before MD and PT was 0.38±0.001 cyc/deg for PSD-95 WT (n = 9, PD 78–149; Fig 3B) and 0.38±0.003 cyc/deg for PSD-95 KO mice (n = 14, PD 93–152; Fig 3A), and thus not different between the genotypes (t-test, p = 0.228). In contrast to nonlesioned mice, daily testing of the PT-lesioned animals after MD did *not* result in an increase in the measured values: the spatial frequency threshold of the optomotor reflex of the open eye was 0.38±0.001 cyc/deg for WT (p = 0.149) and 0.39±0.003 cyc/deg for KO mice (p = 9.989) after 7 days of MD. A similar absence of the experience-enabled enhancement of the optomotor reflex after MD was previously reported for S1-lesioned WT mice [6]. Finally, values were similar in both PSD-95 WT and PSD-95 KO mice after 7 days of PT and MD (p = 0.402, t-test). PSD-95 KO mice without a lesion showed a significant increase of the optomotor reflex by 22% from 0.36±0.01 cyc/deg before MD to 0.44±0.01 cyc/deg after MD (n = 8, PD 70–296; p<0.001, t-test; Fig 3A).

Baseline (before MD and PT) contrast sensitivity values of the optomotor reflex were also not different between PSD-95 WT and PSD-95 KO mice (values at 0.031cyc/deg: WT/KO: 4±0.1 (corresponds to 26% contrast)/4±0.01 (26%), 0.064cyc/deg: 14±0.2 (7%)/14±0.2 (7%), 0.092cyc/deg: 14±0.2 (7%)/14±0.2 (7%), 0.103cyc/deg: 13±0.2 (8%)/13±0.3 (8%); 0.192cyc/deg: 8±0.5 (12%)/8±0.4 (12%), 0.272cyc/deg: 4±0.2 (25%)/5±0.03 (22%); n = 9/14; p>0.05 for every spatial frequency, t-test). Like the spatial frequency threshold, contrast sensitivity thresholds of the open eye did neither increase in PT-lesioned PSD-95 WT (Fig 3F) nor in PSD-95 KO mice (Fig 3E) during MD (p>0.05 for both groups and every spatial frequency, t-test). Hence, contrast sensitivity thresholds of the optomotor reflex of the open eye after 7 days of MD and PT were not different between WT and KO mice (values at 0.031cyc/deg: WT/KO: 4±0.1 (corresponds to 26% contrast)/4±0.01 (27%), 0.064cyc/deg: 14±0.3 (7%)/15±0.4 (7%), 0.092cyc/deg: 14±0.2 (7%)/15±0.4 (7%), 0.103cyc/deg: 13±0.2 (8%)/14±0.4 (7%), 0.192cyc/deg: 9±0.5 (12%)/9±0.5 (12%), 0.272cyc/deg: 4±0.1 (25%)/4±0.03 (22%); n = 9/14; p>0.05 for every spatial frequency, t-test). Notably, nonlesioned PSD-95 KO (Fig 3C) as well as PSD-95 WT (Fig 3D) displayed a significant increase of contrast sensitivity after MD at all measured frequencies (contrast sensitivity before/after MD; for KO (n = 4): 0.031cyc/deg: 3±0.05 (corresponds to 29% contrast)/4±0.1 (23%), 0.064cyc/deg: 12±0.8 (8%)/19±0.7 (5%), 0.092cyc/deg: 11±0.9 (9%)/17±0.7 (6%), 0.103cyc/deg: 10±0.7 (10%)/16±0.7 (6%), 0.192cyc/deg: 6±0.4 (16%)/9±0.3 (11%), 0.272cyc/deg: 3±0.1 (30%)/4±0.1 (23%); for WT (n = 8): 0.031cyc/deg: 4±0.1 (27%)/5±0.05 (22%), 0.064cyc/deg: 15±0.4 (7%)/22±0.3 (5%), 0.092cyc/deg: 14±0.4 (7%)/19±0.4 (5%), 0.103cyc/deg: 13±0.4 (8%)/18±0.4 (8%), 0.192cyc/deg: 7±0.2 (14%)/10±0.5 (10%), 0.272cyc/deg: 4±0.04 (27%)/5±0.1 (22%); at least p<0.01 for every spatial frequency, t-test).

Thus, in contrast to the preserved OD-plasticity in V1, experience-enabled enhancements of the optomotor reflex of the open eye after MD were compromised in both the S1-lesioned PSD-95 KO and PSD-95 WT mice, as previously also reported for S1-lesioned WT (Bl6/J) mice [6].

## Discussion

Our results demonstrate that OD-plasticity in V1 after a cortical stroke lesion in the adjacent S1-area is preserved in PSD-95 KO mice but not in PSD-95 WT mice. In contrast, the experience-enabled enhancement of the optomotor reflex of the open eye after MD was compromised in both PSD-95 KO and PSD-95 WT mice.

We have previously shown that OD-plasticity in V1 of standard cage raised adult mice does not only depend on modality-specific and local nerve cell networks [1, 2], but is also influenced by long-range interactions even from distant brain regions: a small PT-lesion in both S1 or M2 abolished OD-plasticity in V1, indicating that activity changes in the major thalamocortical afferents to V1 are not sufficient to induce OD-shifts in V1 after MD [6, 7]. Notably, two different nonpharmacological interventions that were shown to reduce intracortical inhibition in V1 of adult rodents, namely raising animals in an enriched environment [9] or exposing adult rodents to complete darkness for two weeks [12] did not only restore OD-plasticity in (nonlesioned) old mice but additionally preserved OD-plasticity in V1 even after a small PT-lesion in S1 [9, 12]. In addition, young standard cage raised mice that genuinely display lower intracortical inhibition in V1 compared to older SC-raised animals were also protected against stroke-induced impairments of OD-plasticity [9]. Whether changes in excitatory circuitry may also be beneficial for preserving V1-plasticity after a stroke in S1 was not yet tested.

Since we recently observed that KO mice for the postsynaptic signaling scaffold PSD-95 preserve a high number of AMPA-silent synapses in V1 into adulthood which cause lifelong juvenile-like OD-plasticity despite a normal maturation of the inhibitory tone [20], we wondered whether OD-plasticity in adult PSD-95 KO mice would also persist after a PT-lesion in S1. This was indeed the case. In the PSD-95 KO mice, but not in the PSD-95 WT mice, strong OD-shifts were induced after MD, even in PSD-95 KO mice already beyond PD 110, an age at which OD-shifts in SC-raised WT mice are no longer present [1, 2, 28].

What could be the mechanism underlying the preserved OD-plasticity after stroke in the PSD-95 KO mice? The current literature points towards a protective role of loss-of-PSD-95 function by decoupling excitotoxic signaling. PSD-95 is enriched at the postsynaptic density of glutamatergic synapses and binds e.g. N-Methyl-D-aspartate receptors (NMDAR), other ion channels, and adhesion molecules [29]. PSD-95 links the NMDAR subunit GluN2B to the death-signaling protein neuronal nitric oxide synthase (nNOS) [30]. nNOS catalyzes the production of nitric oxid (NO), a highly neurotoxic signaling molecule. Following an ischemic insult, PSD-95 binds nNOS to GluN2B, thus positioning nNOS in a way that it is more effectively activated by Ca^2+^ influx through the NMDAR ultimately producing NO and enhancing neurotoxicity [31, 32]. Following these insights, *in vivo* studies proved that pharmacological or viral interruption of the GluN2B-PSD-95-nNOS interactions reduces ischemic brain damage and improves neurological scores in both rodents [33–35] and macaques [36, 37]. Moreover, a phase 2 clinical trial was able to demonstrate neuroprotective effects of the drug NA-1 (Tat-NR2B9c), which also disrupts the binding of nNOS to NMDAR by PSD-95 [38]. Thus, disrupting the connection between PSD-95 and excitotoxic signaling can reduce ischemic brain damage and improve neurological scores in rodents as well as in nonhuman primates (reviews [39–41]).

In our lesion model, however, we did not find direct evidence for reduced brain damage. PT-lesions in PSD-95 KO mice were not smaller compared to PSD-95 WT mice. Thus, in contrast to studies addressing the GluN2B-PSD-95-nNOS pathway [33–35, 37], we did not obtain evidence for a reduction in lesion size in PSD-95 KO compared to PSD-95 WT mice. This is most likely due to different lesion models. While we used a small photothrombotically induced lesion in S1, the other studies applied a middle cerebral artery occlusion (MCAO) which typically results in a pronounced penumbra region, which potentially is salvageable if an appropriate treatment is given [42]. Such a penumbra is—however—typically absent after a PT-stroke lesion. Taken together, the increased OD-plasticity that we have observed in V1 of PSD-95 KO mice is most likely not mediated by a reduced lesion size in the PSD-95 KO mice compared to the PSD-95 WT mice.

We rather propose that the increased number of AMPA-silent synapses persisting into adulthood in PSD-95 KO mice represent the substrate which promotes V1-plasticity in both the healthy brain [20] and even after a lesion in S1. Silent synapses in the hippocampus have previously been described as substrates for activity-dependent strengthening of synaptic transmission at excitatory synapses [43, 44]. Therefore, they are most likely beneficial for refinement and reorganization of the cortical network after injury. We recently showed that OD-plasticity was preserved lifelong in V1 of PSD-95 KO mice indicating that high numbers of AMPA-silent synapses prevent the closing of the critical period for juvenile OD-plasticity by preserving a juvenile synaptic state into adulthood [20]. Whether other experimental paradigms of cortical plasticity, such as the long-range influence on OD-plasticity by a distant brain lesion, share similar mechanisms or are distinct had not yet been tested. In fact, our new data unambiguously demonstrate that PSD-95 KO mice showed increased plasticity also in this paradigm: after an S1-lesion, there was preserved OD-plasticity in V1 of the PSD-95 KOs, even when the mice were already at an advanced age, but not in the PSD-95 WT. While this result is phenomenologically similar to our previous observation of preserved OD-plasticity in both young standard-cage raised mice or adult enriched mice after an S1-stroke, the underlying mechanism is clearly different: on the one hand, reduced intracortical inhibition and no changes in AMPA/NMDA excitatory postsynaptic currents (EPSCs) in young or enriched mice [9], and on the other, more silent synapses but normal inhibitory tone in the PSD-95 KO mice [20]. Thus, the same systems level readout can be achieved by clearly differing molecular mechanisms. Since PSD-95 was essential for closing the critical period for juvenile OD-plasticity and knock-down of PSD-95 in the visual cortex of adult WT mice could even restore juvenile-like OD-plasticity, silent synapses are instrumental for network refinement with inhibition having a permissive function [20]. Importantly, the new data show that absence of PSD-95 promotes plasticity also in another experimental paradigm of cortical plasticity.

While PSD-95 KO mice showed preserved OD-plasticity in V1 after the S1-lesion, experience-enabled improvements of both the spatial frequency and the contrast sensitivity threshold of the optomotor reflex of the open eye after MD were—however—not observed. Such a dissociation between OD-plasticity in V1 and experience-dependent changes of the optomotor reflex after MD has already been reported after ibuprofen treatment in stroke-lesioned standard-cage raised mice [6], after enriched environment housing and a stroke lesion in S1 [9], and after a distant PT-lesion in M2 [7]. The diverging effects of the PT-lesion on OD-plasticity vs. experience-enabled enhancement of the optomotor reflex [45] in the PSD-95 KO mice (preserved vs. abolished) adds to the evidence that distinct neuronal subsystems underlie these two forms of visual plasticity as suggested before [6, 46]. The experience-enabled enhancements of the optomotor reflex of the open eye after MD are restricted to the monocular visual field, despite the dependence of the plasticity on binocular interactions [45]. In contrast, OD-shifts occur in the binocular region of V1 [22, 23, 47]. Moreover, OD-plasticity beyond the critical period mainly takes place in superficial cortical layers [48–50], while the enhancement of the optomotor response involves the cortical control of the accessory optic system triggering the reflex, presumably from deep-layer efferents [45]. Furthermore, there is accumulating evidence that stroke-induced inflammatory processes contribute to the impaired experience-enabled reflex enhancements, but not to OD-plasticity. While anti-inflammatory treatment with ibuprofen rescued the visual enhancements after PT to control levels, it had no beneficial effect on OD-plasticity [6]. Supporting this idea, EE-housing reduced the increased inflammation level after stroke in rats [51] and the enhancement of the optomotor reflex after MD in stroke lesioned mice was at least partially preserved in EE-raised mice [9]. Since optomotor enhancements were not present in PSD-95 KO mice, the plasticity promoting effect of PSD-95 is likely not caused via reducing intracortical inflammation.

In conclusion, knocking-out PSD-95 has a plasticity promoting effect on V1-plasticity after a stroke in S1, but does not rescue experience-enabled improvements of spatial vision of the open eye after MD. Our present findings are very important in showing that plasticity promoting effects in V1 after a stroke in S1 can not only be induced by lowering intracortical inhibition [9, 12] but also by modifying excitatory circuitry. In particular, a lower AMPA-NMDA EPSC ratio and an increased number of AMPA silent synapses—as present during the critical period for juvenile OD-plasticity in V1 of WT mice or lifelong in V1 of PSD-95 KO mice [20]–can not only enable a lifelong and juvenile-like OD-plasticity in nonlesioned animals but also protect against impairments of V1-plasticity induced by a distant stroke lesion.
